# Efficacy of Additional Corticosteroids After Dexamethasone Treatment for Moderate to Severe COVID-19: An Observational Study

**DOI:** 10.7759/cureus.43179

**Published:** 2023-08-09

**Authors:** Yosuke Fukuda, Kaoru Mochizuki, Miharu Ijichi, Tetsuya Homma, Akihiko Tanaka, Hironori Sagara

**Affiliations:** 1 Department of Medicine, Division of Respiratory Medicine and Allergology, Yamanashi Red Cross Hospital, Fujikawaguchiko-machi, JPN; 2 Department of Medicine, Division of Respiratory Medicine and Allergology, Showa University School of Medicine, Tokyo, JPN

**Keywords:** corticosteroids in covid-19, sars-cov-2, acute hypoxemic respiratory failure, organizing pneumonia, covid-19

## Abstract

Background

Previous studies have demonstrated dexamethasone (DEX)'s efficacy for coronavirus disease 2019 (COVID-19). In contrast, patients with residual lung field shading and symptoms after DEX treatment have been observed, and the efficacy of additional corticosteroids (AC) is unknown.

Objectives

We aimed to investigate the efficacy of AC in patients with COVID-19 with residual respiratory symptoms or who required oxygen therapy or invasive mechanical ventilation after DEX treatment.

Methods

This was a single-center, retrospective observational study including 261 patients with community-onset COVID-19, aged ≥ 18 years, admitted to our hospital between March 1, 2020, and May 31, 2021. Finally, 34 patients were included in the study who met all four of the following criteria: (1) required oxygen therapy or invasive ventilation, (2) were treated with DEX, (3) had residual shading on chest imaging after DEX treatment, or (4) had unimproved respiratory symptoms or oxygen saturation < 90%. We reviewed the medical records and clinical courses of 14 patients who received AC therapy (AC group) and 20 patients who did not (non-additional corticosteroids or NC group).

Results

The 90-day mortality rate was 35.7% in the AC group and 25.0% in the NC group. There was no statistically significant difference between the two groups (p = 0.797). In addition, there was no difference between groups in the proportion of patients who required oxygen therapy at discharge (64% vs. 35%, p = 0.162). The time from the end of DEX therapy to discharge was significantly longer in the AC group (median 7.5 vs. 33 days, p = 0.019). Regarding serious adverse events, infection was statistically more common in the AC group than in the NC group (p = 0.005).

Conclusions

AC after DEX treatment does not improve clinical outcomes and may prolong hospital stay.

## Introduction

By March 2023, coronavirus disease 2019 (COVID-19) caused more than 6.8 million deaths worldwide [1]. Among patients infected with COVID-19, 80% have mild disease, while approximately 5% develop shock and multiple organ failure, with an overall mortality rate of 2.3% [2]. The in-hospital mortality rate of COVID-19 was 14%, while those of the influenza virus, a typical virus that causes viral pneumonia, and severe acute respiratory syndrome coronavirus 2, were 6% [3]. This trend of higher mortality in COVID-19 compared to influenza was reportedly more pronounced in patients older than 50 years and younger than 18 years [4]. Thus, COVID-19 is as much or more of a threat to humanity than any current endemic or circulating virus.

There are a few established treatment options for critical patients with COVID-19, one of which is corticosteroids, which have potent anti-inflammatory effects. The RECOVERY trial conducted in the UK examined the efficacy of dexamethasone (DEX) treatment for COVID-19 [5]. The results showed a 28-day mortality rate of 22.9% in the DEX group compared to 25.7% in the standard-of-care group without DEX, especially in patients who received invasive mechanical ventilation [5]. In the CoDEX trial [6], a multicenter randomized control trial to evaluate the efficacy of DEX for patients with COVID-19 requiring ventilator management, ventilator-free days were found to be significantly higher in the DEX group than in the standard care group. The results of these studies support the usefulness of SC for the treatment of COVID-19.

However, the optimal duration and dosage of corticosteroids are still unknown. We often encountered patients with respiratory failure and clinical symptoms that persisted after the prescribed duration of corticosteroid therapy in the clinical setting. In addition, delayed viral excretion was reported in pneumonia caused by viruses that were prevalent in the past, such as Middle East respiratory syndrome coronavirus and influenza [7,8].

Therefore, we aimed to investigate the efficacy of additional corticosteroids (AC) in patients with COVID-19 who had residual respiratory symptoms or required oxygen therapy or invasive mechanical ventilation after DEX treatment.

## Materials and methods

Study design and participants

**Figure 1:**
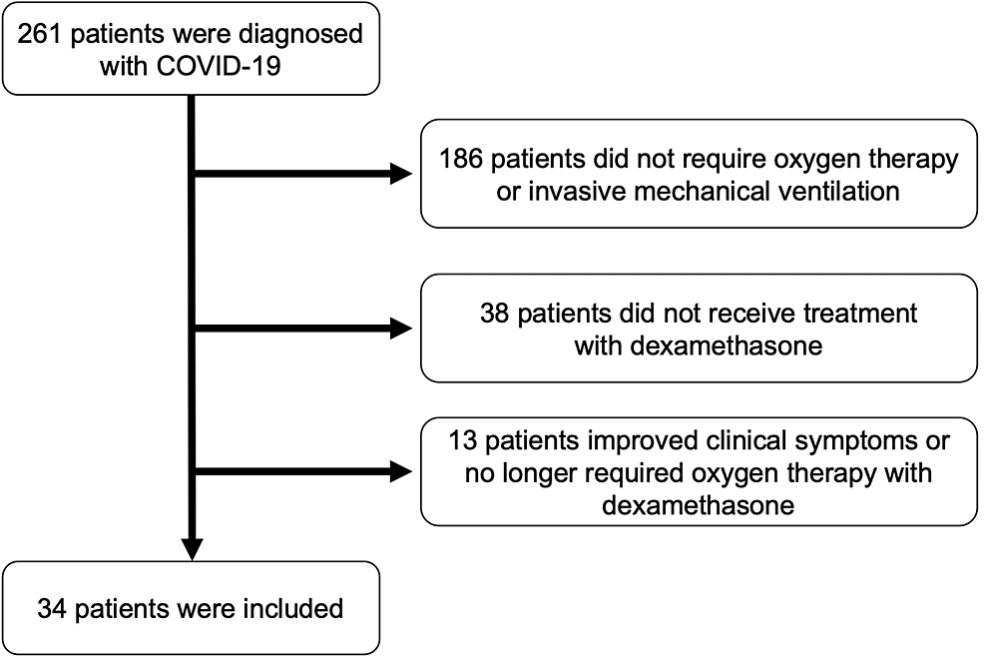
Study flowchart Of the 261 coronavirus disease 2019 (COVID-19) patients screened, 227 were excluded according to the criteria described in the methods section, and 34 were included.

This study was an observational study conducted at a single center. We retrospectively reviewed the medical records of 261 patients aged 18 years and older with community-onset COVID-19 from March 1, 2020, to May 31, 2021. We confirmed the diagnosis of COVID-19 based on a positive polymerase chain reaction test by nasal swab. Among all patients, the following were excluded: 186 patients who did not require oxygen therapy or invasive mechanical ventilation, 38 patients who did not receive DEX treatment, and 13 patients whose symptoms improved or patients who no longer required oxygen therapy after a maximum of 10 days of treatment with a DEX dose of intravenous 6.6 mg/day. Finally, 34 patients were included (Figure 1). After the completion of DEX treatment, the patients were divided into two groups: 20 patients comprised the AC group, and 14 patients comprised the non-additional corticosteroids (NC) group. All patients in both groups were followed up for at least 28 days. Serious adverse effects were examined during the observation period, including infection, thrombosis, bleeding, acute renal failure, pneumothorax, and cardiac complications. The need for additional corticosteroid treatment was based on the consensus of multiple respiratory medicine and intensive care medicine specialists. All data were fully anonymized prior to access.

Statistical analyses

Statistical analyses were performed using JMP software (Version 15; SAS Institute Japan Ltd., Minato-ku, Tokyo, Japan). Continuous and categorical variables were presented as medians (interquartile ranges) and numbers (percentages), respectively. The validity of the normal distribution was assessed using the Shapiro-Wilk test. Fisher's exact test and 95% confidence intervals (CIs) were used to test the differences between categorical variables; the Mann-Whitney U test was used for continuous variables. We excluded points that exceeded 1.5 times the upper and lower quantile range as outliers. Survival curves were estimated using the Kaplan-Meier method, followed by the log-rank test. P values less than 0.05 were considered statistically significant. All data were not fully anonymized before we accessed them.

Ethics approval

This study was conducted in accordance with the Declaration of Helsinki and was approved by the Showa University Ethics Committee (approval number: 21-093-B). After publishing a notice stating that this research would be based on the patient's clinical information on the website of the Showa University Ethics Committee, we obtained informed consent in the form of an opt-out. If the patient was under 20 years of age, consent was obtained from the parents or guardians.

## Results

The median age of the AC group was 76.0 years, compared to 65.5 years in the NC group. There was no significant difference in the sex ratio or incidence of comorbidities between the two groups (Table 1). At the end of the DEX treatment, the mean platelet count was statistically significantly lower in the AC group than in the NC group. However, there were no significant differences in the other parameters (Table 2). 

**Table 1:** Clinical characteristics of all patients AC, additional corticosteroids; BMI, body mass index; COPD, chronic obstructive pulmonary disease; NC, non-additional corticosteroids

	AC group (n = 14)	NC group (n = 20)	p value
Age, year	76.0 (70.5-83.2)	65.5 (58.2-77.7)	0.049
Sex, male/female, n	5/9	6/14	1.000
BMI, kg/m^2^	23.1 (21.0-25.2)	25.0 (21.6-27.0)	0.420
Smoking status, Former or Current, n	6 (42.8)	11 (55.0)	0.728
Respiratory support received at the end of dexamethasone, n (%)			
Oxygen	12 (85.7)	17 (85.0)	1.000
Invasive mechanical ventilation	7 (50.0)	7 (35.0)	0.486
Other treatment during hospitalization, n (%)			
Remdesivir	10 (71.4)	15 (75)	1.000
Favipiravir	3 (21.4)	4 (20.0)	1.000
Tocilizumab	2 (14.2)	4 (20.0)	1.000
Baricitinib	1 (7.1)	2 (10.0)	1.000
Extracorporeal membrane oxygenation	2 (14.2)	2 (10.0)	1.000
Comorbidities, n (%)			
Hypertension	10 (71.4)	12 (60.0)	0.717
Diabetes mellitus	5 (35.7)	5 (25.0)	0.704
Dyslipidemia	4 (28.5)	6 (30.0)	1.000
Hyperuricemia	1 (7.1)	3 (15.0)	0.627
Asthma	2 (14.2)	1 (5.0)	0.555
COPD	2 (14.2)	2 (10.0)	1.000
Interstitial lung disease	1 (7.1)	0 (0)	0.411
Autoimmune disease	3 (21.4)	1 (5.0)	0.283
Chronic kidney disease	1 (7.1)	1 (5.0)	1.000
Malignancy	3 (21.4)	0 (0)	0.060
Chronic heart disease	4 (28.5)	6 (30)	1.000

**Table 2:** Difference in blood examination between the two groups at the end of intravenous dexamethasone AC, additional systemic corticosteroids; ALP, alkaline phosphatase; ALT, alanine aminotransferase; APTT, activated partial thromboplastin time; AST, aspartate aminotransferase; CRP, C-reactive protein; GTP, glutamyl transpeptidase; KL-6, Krebs von den Lungen-6, NC, non-additional systemic corticosteroids; PT, prothrombin time; SP, surfactant protein

	AC group (n = 14)	NC group (n = 20)	p value
White blood cell, /μL	11700 (7950-12750)	9400 (6700-13850)	0.624
Neutrophil count, /μL	10390 (6170-11960)	7505 (5542-11175)	0.391
Lymphocyte count, /μL	645 (417-1015)	1085 (595-1750)	0.089
Hemoglobin, g/dL	12.3 (10-13.5)	12.7 (11-12.9)	0.674
Platelet count, ×10^4^/μL	20.0 (11.2-30.5)	30.6 (18.7-37)	0.030
PT	1.26 (1.17-1.36)	1.13 (1.04-1.22)	0.061
APTT	44.1 (31.6-65.5)	36.1 (28.6-56.8)	0.410
Fibrinogen, mg/dL	493 (319-758)	476 (361-633)	0.877
D-dimer, ng/mL	4.00 (1.39-5.92)	1.87 (1.10-5.10)	0.431
Total protein, g/dL	5.5 (5-5.9)	5.5 (5.3-6.2)	0.558
Albumin, g/dL	2.40 (2.35-2.65)	2.65 (2.17-3.1)	0.267
Urea nitrogen, mg/dL	22 (17.2-42)	22.4 (17.7-39.6)	0.888
Creatinine, mg/dL	0.67 (0.61-0.83)	0.77 (0.66-1.12)	0.151
Uric acid, mg/dL	3.8 (2.8-4.3)	5 (3.3-6.8)	0.149
AST, U/L	23.5 (19-33.7)	29 (20.5-37.5)	0.301
ALT, U/L	22 (14.5-49.2)	27.5 (20-57.7)	0.353
Lactate dehydrogenase, U/L	371 (255-451)	300 (226-437)	0.624
ALP, U/L	79 (64-104)	71 (65-84.5)	0.506
γ-GTP, U/L	42 (30-76)	61 (36-117)	0.258
Creatine kinase, U/L	40 (22-120.5)	36 (22-108)	0.841
Sodium, mEq/L	139.1 (136.1-143.5)	139.2 (136.6-143.7)	0.930
Potassium, mEq/L	4.3 (3.9-4.4)	4.1 (3.9-4.6)	0.712
CRP, mg/dL	2.87 (1.13-5.49)	2.04 (0.42-6.74)	0.611
Ferritin, ng/mL	839 (392-1180)	768 (226-1608)	0.779
KL-6, U/mL	1078 (788-1597)	1013 (245-2143)	0.748
SP-D, ng/mL	613 (149-1078)	125 (107-459)	0.182
SP-A, ng/mL	176 (176-176)	55.1 (22.5-220)	0.654

**Figure 2:**
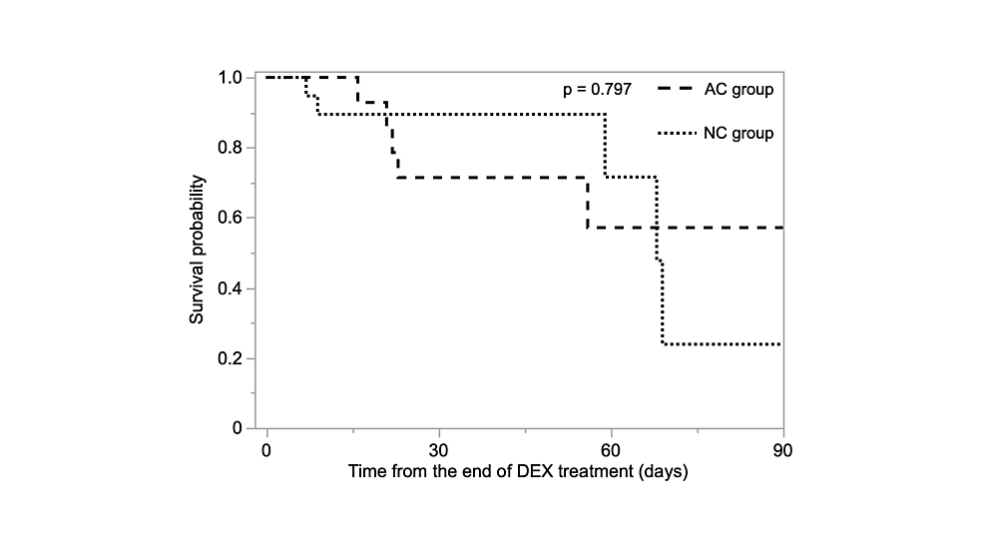
Kaplan-Meier survival curves for the 90-day survival with or without additional corticosteroids to DEX There was no statistically significant difference in the 90-day mortality between the two groups (p = 0.797). AC, additional corticosteroids; DEX, dexamethasone; NC, non-additional corticosteroids

The 90-day mortality rate was 35.7% in the AC group and 25.0% in the NC group. There was no statistically significant difference between the two groups (p = 0.797, Figure 2). Regarding the other clinical outcomes, the proportion of patients discharged within 28 days of DEX treatment initiation was significantly higher in the NC group than in the AC group (p = 0.030, Table 3). Furthermore, the time from onset to discharge, time from COVID-19 diagnosis to discharge, time from admission to discharge, and time from the end of DEX treatment to discharge were all statistically significantly longer in the AC group (p = 0.014, p = 0.012, p = 0.011, and p = 0.018, respectively).

**Table 3:** Difference in clinical outcomes between the two groups AC, additional corticosteroids; DEX, dexamethasone; NC, non-additional corticosteroids

Outcome	AC group (n = 14)	NC group (n = 20)	p value
Discharge in 28 days from the end of DEX, n (%)	2 (14.2)	11 (55.0)	0.030
Death during hospitalization, n (%)	5 (35.7)	5 (25.0)	0.704
Need oxygen at discharge, n (%)	9 (64.2)	7 (35.0)	0.162
Length, days			
From onset to discharge	49 (33.2-91)	24.5 (21-68.2)	0.014
From diagnosis to discharge	48 (30.7-89.7)	20 (17.2-66)	0.012
From admission to discharge	43 (29.2-85.5)	17.5 (15-64)	0.011
From the end of DEX to discharge	33 (21.7-73)	7.5 (6-57.7)	0.018

Regarding serious adverse events, infection was statistically more common in the AC group than in the NC group (p = 0.005, Table 4). While four patients were diagnosed with ventilator-associated pneumonia, two patients with pneumocystis pneumonia, two patients with invasive pulmonary aspergillosis, one patient with hospital-acquired pneumonia in the AC group, and three patients were diagnosed with ventilator-associated pneumonia in the NC group. Thrombosis, bleeding, acute renal failure, pneumothorax, and cardiac complications were not statistically significant between the two groups.

**Table 4:** Difference in serious adverse events between the two groups AC, additional corticosteroids; NC, non-additional corticosteroids

Serious adverse events	AC group (n = 14)	NC group (n = 20)	p value
Infection	9	3	0.005
Thrombosis	2	0	0.162
Bleeding	3	2	0.627
Acute renal failure	1	6	0.197
Pneumothorax	0	0	1.000
Cardiac complications	6	5	0.457

## Discussion

This study is the first to demonstrate that corticosteroids, in addition to DEX, did not improve mortality and prolonged the length of hospital stay in patients with COVID-19. Furthermore, we found that infection was a significantly more common serious adverse effect in the AC group compared to the NC group.

In the RECOVERY trial, approximately 25% of the patients treated with DEX died at 28 days [5]. In a randomized controlled trial that compared the effects of 6 mg/day and 12 mg/day of DEX on patients with severe COVID-19, 12 mg/day of DEX did not improve mortality at 28 days [9]. This finding is consistent with the results of our study in that ACs did not improve mortality in patients with COVID-19. We would also expect mortality rates to vary depending on whether anti-interleukin-6 receptor antibodies [10,11], Janus kinase inhibitors [12,13], or antiviral drugs such as remdesivir [14,15] were used in combination. Although a meta-analysis is currently underway [16], we may also need to refer to evidence from other respiratory viral infections to determine the appropriate dose of corticosteroids for the treatment of COVID-19.

Our findings indicate that AC after DEX treatment prolonged the length of hospital stay. Recently, several studies comparing the efficacy of DEX and methylprednisolone (MP) in hospitalized patients with COVID-19 have been reported [17,18]. In a randomized controlled trial conducted in Iran, the group treated with 2 mg of MP per kg once daily, tapered to half the dose every five days, had a shorter length of hospital stay compared to the group treated with 6 mg of DEX daily for 10 days (7.43 ± 3.64 days vs. 10.52 ± 5.47 days, p = 0.015) [17]. Similarly, patients treated with 50 mg of oral prednisone for 14 days after three days of high-dose (250-500 mg/day) MP showed significant improvement in blood biomarkers such as C-reactive protein and lactate dehydrogenase compared to the DEX group [18]. Although it was unclear whether these studies reflect a difference in the pharmacological effects of DEX and MP, they indicate that a longer-term treatment strategy using different types of corticosteroids, other than DEX for COVID-19, might shorten the length of hospital stay.

In this study, there was a significantly higher incidence of infection in the AC group than in the NC group. Infection is a significant adverse event associated with corticosteroid use. COVID-19 is associated with bacterial and fungal infections in approximately 8% of patients [19] and is often critical. Previous reports have shown no statistical difference in the proportion of serious infections associated with different doses of steroids in patients with COVID-19 [9,12]. While we estimated that the median age of the AC group was 76 years in this study, the median age of the population was 55 [12] or 65 years [9] in previous studies. This fact may have potentially influenced their susceptibility to infection.

**Figure 3:**
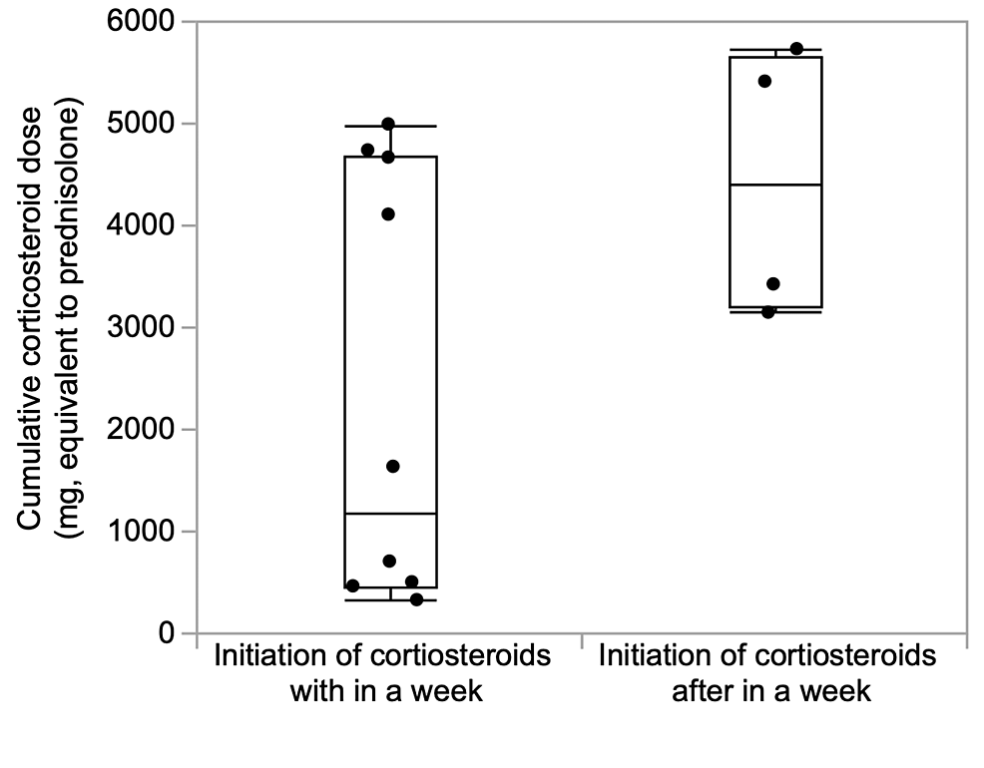
Cumulative corticosteroid dose according to the timing of additional corticosteroid treatment initiation There was no significant difference in the timing of starting additional corticosteroids within or after a week (n = 14; p = 0.089).

The strength of this study is that it is the first to prove that corticosteroids added to DEX did not benefit patients with moderate to severe COVID-19. It is not clear why the AC treatment did not improve clinical outcomes, but we hypothesized that this was partially due to the significant increase in infections with the ACs in our study. There are some limitations to this study. First, it was a small, single-center study; the sample size may have been insufficient to accurately examine the benefit of AC treatment after DEX. Second, the timing of the initiation of AC treatment and the total dosage was not uniform; there was no difference in the cumulative total corticosteroid doses between patients who started additional SC within one week after the end of DEX treatment and patients who started after one week (Figure 3). Third, it is unclear whether the conditions of the groups of included patients were uniform. It is often difficult to determine whether the patient’s condition after DEX treatment is the initial stage of sequelae [20], secondary organizing pneumonia [21], or acute respiratory distress syndrome [22], which is refractory to initial treatment. In a clinical trial of interstitial lung disease after COVID-19, it was reported that 4.8% of patients had interstitial lung disease with functional impairment four weeks after discharge [23]. Corticosteroids in these patients accelerated the recovery of respiratory function and resulted in radiological improvement [23]. A randomized controlled trial of corticosteroid treatment strategies for secondary organizing pneumonia after COVID-19 is underway at a university hospital in Spain [24]. 

## Conclusions

In conclusion, this study found that AC after DEX treatment did not improve mortality or length of hospital stay in patients with moderate to severe COVID-19. Furthermore, the AC group induced significantly more infections than the control group. These results suggest that, while DEX is effective for COVID-19 with respiratory failure, if DEX does not completely recover the respiratory status caused by COVID-19, ACs may not be effective, regardless of the pathology. This is important in terms of the optimal dose and duration of corticosteroids for COVID-19. Consolidating findings in severe viral pneumonia other than SAR-CoV-2, such as influenza, in addition to findings in the sequelae of COVID-19 and secondary organizing pneumonia, we believe that further research will complement the current lack of understanding of the respiratory pathogenesis after DEX treatment for severe COVID-19.
